# Efficient Energy Conversion and Storage Based on Robust Fluoride‐Free Self‐Assembled 1D Niobium Carbide in 3D Nanowire Network

**DOI:** 10.1002/advs.201903680

**Published:** 2020-04-06

**Authors:** Sin‐Yi Pang, Weng‐Fu Io, Lok‐Wing Wong, Jiong Zhao, Jianhua Hao

**Affiliations:** ^1^ Department of Applied Physics The Hong Kong Polytechnic University Hong Kong P. R. China

**Keywords:** 2D materials, electrocatalysts, flexible batteries, MXenes, nanowires

## Abstract

Owing to their high robustness and conductivity, 2D transition metal carbides and nitrides known as MXenes are considered as a promising material class for electrochemical catalysis, energy conversion, and storage applications. Nevertheless, conventional hazardous fluoride‐based synthesis routes and the intense intralayer bonding restrict the development of MXenes. Herein, a fluoride‐free, facile, and rapid method for synthesizing self‐assembled 1D architecture from an MXene‐based compound is reported. The MXene nanowire (NW) not only provides a robust connection to the flexible substrate but also effectively increases the electrochemically active surface area. The kinetics‐favorable structure yields a boosted performance for the hydrogen/oxygen evolution reaction and the intake of the zinc ion. The 1D NW based on MXene compound maintains high stability in a quite low overpotential of 236 mV for 24 h without detachment from the substrate and manifests an exceptional high‐power density of 420 W kg^−1^ over 150 cycles as a flexible aqueous zinc ion battery. This work paves a novel and non‐toxic synthesis method for the 1D nanofiber structure from MXene composition and demonstrates its multifunctional applications for energy conversion and storage.

## Graphical abstract

With the facile technique in alkali hydrolysis and HF‐free electrochemical etching, MXene nanowire is fabricated in a shortened etching time without the utilization of hazardous HF acid. The MXene NW@3D substrate not only renders a boosted kinetics and shortened diffusion length, but also offers a stable hydrogen evolution activity and flexible zinc ion battery application even during the deformation progress.

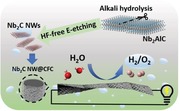

2D materials have drawn tremendous attention and extensive investigations for emerging applications in energy, electronics, and optoelectronics have been conducted.^[^ ^1^, ^2^ ^]^ In particular, 2D transition metal carbides and nitrides (MXenes) are considered as emerging energy materials. Enjoying their endowments in the high hydrophilic surface, porosity, and conductivity, MXenes possess widespread applications in battery, electrocatalyst, supercapacitor, and biochemistry.^[^ ^3^, ^4^, ^5^, ^6^, ^7^ ^]^ MXenes are usually described by a chemical formula of M*_n_*_+1_X*_n_*T*_x_* (*n* = 1, 2, and 3), where M represents an early transition metal (such as Ti, Nb, Cr, and V.), X stands for the C and/or N element, and T*_x_* is the terminational groups, for instance, —OH, —O, —Cl, and —F.^[^ ^8^ ^]^ With high flexibility on enriched functionalization, MXenes accommodate a widened compatibility toward various hybrid system coupled with a favorable mechanic strength up to 55 GPa.^[^ ^9^ ^]^ Based on the theoretical calculation, the tuning on the surface termination renders a modifiable bandgap from metallic to semiconducting for MXenes.^[^ ^10^ ^]^ Driven by their tunable properties, MXenes have also demonstrated emerging electronic and optical device applications.^[^ ^10^ ^]^ Until now, more than 20 species of MXenes have been synthesized by chemical etching with the fluoride‐etchant.^[^ ^11^ ^]^ However, restriction is found in some MAX systems which are not able to synthesize the MXene via either HF etching or molten salt route.^[^ ^12^ ^]^ Meanwhile, HF reagent is a harmful poison with the hazard of causing systemic toxicity in the human body or direct fatality. Furthermore, unstable HF‐species in HF‐etched MXene may inhibit the stability of electrochemical reaction upon electrolyte/electrode interaction.^[^ ^13^ ^]^ Nonetheless, only a few studies regarding the preliminary HF‐free synthesis of titanium carbides (Ti_2_C or Ti_3_C_2_) have been reported.^[^ ^14^, ^15^, ^16^, ^17^ ^]^ It is essential that the properties of these emerging fluoride‐free MXenes and extended strategy to other MAX phase material are explored. To date, the enhancement of the surface functional group engineering was demonstrated in the HF‐free Ti_3_C_2_. With the enriched —O/OH terminations, the HF‐free MXene exhibits a high volumetric capacitance as supercapacitor.^[^ ^17^ ^]^ As a result of simultaneous intercalation and etching process, HF‐free MXene takes advantage in a higher yield of few‐layered 2D products without extra delamination procedure.^[^ ^16^ ^]^ V_2_C, Ti_2_C, and Cr_2_C have demonstrated a good Zn^2+^ ion intake ability as a rechargeable aqueous zinc ion battery (AZIB) with high retention that is superior to the HF‐etched MXenes.^[^ ^14^, ^18^ ^]^

In the family of the ordered double MXene, niobium carbide (Nb_2_C) holds its specificity in metallic properties with a flexible combination of functional groups. Compared to other reported double layered MXenes, Nb_2_C possesses an excellent conductivity with almost zero or small bandgap that also furnishes its prominence which is distinct from traditional 2D materials.^[^ ^10^, ^19^, ^20^ ^]^ In the merit of good biocompatibility, near‐infrared (NIR) adsorption, and high photothermal conversion performance,^[^ ^21^ ^]^ Nb_2_C is chosen as a promising candidate for both in vitro and in vivo photothermal/bio‐detection applications.^[^ ^6^, ^7^ ^]^ On the other hand, Nb_2_C, which exhibits a good ion intake ability, is vastly used as a cathode for metal ion battery.^[^ ^3^, ^22^ ^]^ Unfortunately, the common synthesis route for MXenes usually requires a highly concentrated HF solution and relatively long reaction time (50% conc., 90 h).^[^ ^3^ ^]^ Furthermore, the —F terminated MXenes are likely to release HF during the hydrogen evolution reaction (HER) process and the toxicity of fabrication process hampers the development of MXene.^[^ ^13^ ^]^ Moreover, the restacking of 2D MXene due to the van der Waals (vdW) force interaction worsens the surface kinetics and the ion exchange process, resulting in poor electrochemical energy storage for the MXene electrode.^[^ ^22^ ^]^

The utilization of rational architecture design and carbon‐based backbones is essential for fabricating an aggregation‐resistant open structure to feature a fast surface kinetics.^[^ ^4^, ^23^, ^24^ ^]^ Noted that MXene compound based nanofiber,^[^ ^25^ ^]^ crumped devices,^[^ ^23^ ^]^ and macropores^[^ ^24^ ^]^ have been successfully fabricated and demonstrated their outstanding applications. It is attributed to their enhanced conductivity and ion accessibility. In sharp contrast to 2D MXene, 1D/3D architecture from MXene composition features a remarkable enhancement toward electrochemical activity due to its enriched basal active sites, specifically beneficial to HER.^[^ ^4^, ^25^ ^]^ In addition to the dimensional engineering on MXenes, the assistance of porous carbon‐based substrates/backbones can effectively prevent from vdW force‐driven compact restacking.^[^ ^22^ ^]^ Carbon fiber, carbon fiber nanotubes (CNT), and carbon fiber cloth (CFC) are widely used in the fabrication of electrochemical device as supercapacitor and zinc–air battery combining other 2D materials.^[^ ^26^, ^27^ ^]^ Fiber/cable like supercapacitor also renders a novel and fast energy storage option to the emerging wearable device,^[^ ^28^, ^29^ ^]^ and the conjugation of MXene‐polymer features self‐healing function to the MXene supercapacitor.^[^ ^30^ ^]^ The carbon‐based substrate offers a 3D network for 2D materials and nanoparticle adsorption, thus decreasing the total resistance of the system.^[^ ^31^ ^]^

Hence, the overall energy storage and conversion activity of MXenes are expected to be improved by combining a suitable substrate and rational design of MXene. Herein, we adopt a modified electrochemical etching (E‐etching) method to synthesize MXenes with a kinetics‐favored architecture and significantly shorten the synthesis time (4 h) by 22‐fold, compared to the traditional synthesis route. By conjugating the porosity‐rich CFC and niobium carbide nanowires (NW), the multifunctional energy devices enable fast kinetics toward HER and oxygen evolution reaction (OER), as well as Zn^2+^ ion intakes. Taking advantage of the sharp reduction of internal resistance, the Nb_2_CT*_x_* NW exhibits a quite low overpotential toward HER of 322 mV, which is close to the theoretical value.^[^ ^32^ ^]^ The boosting effect on the electrocatalytic activity of the 3D‐Nb_2_CT*_x_* NW is attributed to transitional metal ions (Fe, Ni, Co) promotor, lowering of the O—H bonding strength of the H_2_O molecules.^[^ ^33^ ^]^ Moreover, the overpotential of Co^3+^@3D‐Nb_2_CT*_x_* NW is the lowest in the reported literatures for HER and OER activity. The novel design also provides a good Zn^2+^ intake for aqueous ZIB. The fabricated flexible battery can maintain excellent retention of 100% over 150 cycles with a stable power delivery during deformed condition.

The HF‐free synthesis process for Nb_2_CT*_x_* NW is schematically depicted in **Figure** **1**. There is a two‐step etching process included in the synthesis procedure: 1) hydrolysis and 2) 3D electrode thermo‐assisted E‐etching for selective removal of the Al layer from the MAX phase precursor, as proposed in the following equation:

(1) $$\begin{array}{l} {\text{Nb}}_{2}\text{AlC}+{\text{yCl}}^{-}+\left(2x+z\right){\text{H}}_{2}\text{O}\to {\text{Nb}}_{2}\text{C}{\left(\text{OH}\right)}_{2x}{\text{Cl}}_{y}{\text{O}}_{z}+{\text{Al}}^{3+}\\ +\left(x+z\right){\text{H}}_{2}+\left(y+3\right){\text{e}}^{-} \end{array}$$

The MAX phase Nb_2_AlC precursor was first hydrolyzed by 6 m KOH solution. With the assistance of heating and intense stirring, the MAX phase precursor generated crevices at the surface Ti—C sites and subsequently cleaved into small pieces,^[^ ^25^ ^]^ resulting in a reduced lateral size from 10–30 to 1–5 µm (Figure S1a,b, Supporting Information). Considering the investigation of etching time–depth relationship from a previous work,^[^ ^14^ ^]^ a shorter etching time was utilized to allow a “shred effect” of nanowire from the etched MAX‐MXene composite with the assistance of ultrasonication. High‐quality Nb_2_CT*_x_* nanosheet (NS) and NW were made by E‐etching method (details as seen in Experimental Section). The cyclic voltammetry (CV) curve was recorded for determining the appropriate selectively Al etching voltage, as plotted in Figure S1c, Supporting Information. The CV curve shows two board peaks with a pair of vertex points at 0.45 V and 1.20 V, which are attributed to the reaction between the Cl^−^ ion to the Al layer and the Nb layer, respectively. The two peaks obey the two‐step process route: The first one is selective etching of the “A” layer (stage 1) and the second one is complete etching of two metal layers (stage 2) from its parent material.^[^ ^14^, ^22^ ^]^ The appropriate voltage for E‐etching for 4 h at 1 V is further confirmed by transmission electron microscope (TEM) image and the linear sweeping voltammetry (LSV) of the E‐etched MXene (**Figure** **2**a; Figure S1d, Supporting Information). Subsequently, insufficient etching depth was found in the SEM images (Figure S1e, Supporting Information) with an etching voltage of 0.5 V, whereas successful etching was observed in Figure S1f, Supporting Information, at an etching voltage of 1.0 V, holding consistency with electrochemical, structural, and elemental analytical results (Table S1, Supporting Information).

**Figure 1:**
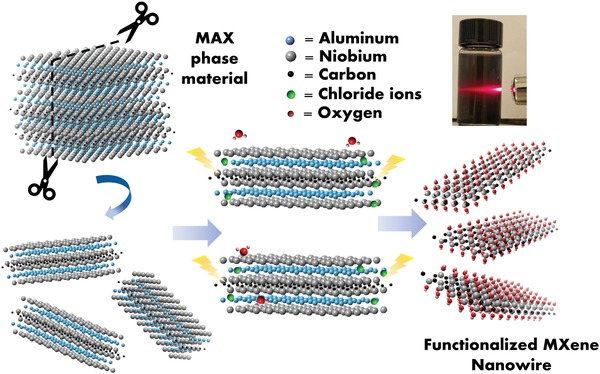
Schematic of the Nb_2_CT*_x_* NW synthesis process via hydrolysis and HF‐free E‐etching method. The inset digital photo shows clear Tyndall scattering effect of the water‐dispersed MXene colloids.

**Figure 2:**
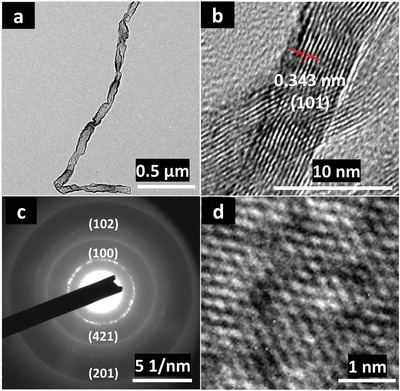
Morphology study of Nb_2_CT*_x_* NW. a) The TEM and b) HRTEM image of the E‐etched Nb_2_CT*_x_* NW. c) The SAED pattern of the high quality Nb_2_CT*_x_* NW. d) The fast Fourier transform filtered HRTEM image shows the hexagonal atomic arrangement of the Nb_2_CT*_x_* NW, where the defects (dark sites) were possibly attributed to the hydrolysis process.

The structure and phase of the Nb_2_CT*_x_* NW were examined by Raman spectroscopy, X‐ray diffraction (XRD) and X‐ray photoelectron spectroscopy (XPS). The Raman spectrum (Figure S2a, Supporting Information) of the sample illustrates distinct vibration peaks at 214, 277, 388, and 493 cm^−1^, which are deviated from that for the pristine MAX phase precursor;^[^ ^7^, ^34^ ^]^ while the peak shift from 400 to 388 cm^−1^ is probably due to the vanished Al—C bonding.^[^ ^35^ ^]^ Two characteristic broad peaks locating at 1332 and 1582 cm^−1^ are assigned to the D and G bands of the amorphous carbon on the surface of the MXene, respectively.^[^ ^36^ ^]^ The XRD curves indicate the removal of Al layer from the MAX phase precursor which leads to an increase in the *c*‐lattice parameter from 1.39 to 1.52 nm corresponding to the peak shift from 12.7° to 11.6°,^[^ ^22^, ^34^ ^]^ as shown in Figure S2b, Supporting Information. Notably, a broadened XRD peak of ≈25°, which is commonly observed in nanoscale MXene, might possibly be attributed to the delaminated nature of the MXene .^[^ ^37^, ^38^ ^]^ XPS survey demonstrates the vanished Al binding energy signal on 70 eV (Figure S2c, Supporting Information), revealing the success of selective E‐etching of Al from its MAX phase. Figure S2d, Supporting Information, manifests high‐resolution XPS on Nb 3d, and unveils the presence of Nb(V)‐O and functionalized Nb_2_C bonding, suggesting the formation of Nb_2_C(O)/(OH) and Nb_2_O_5_ after the E‐etching.^[^ ^22^ ^]^

To further study and characterize the morphology and lattice properties of the pure Nb_2_CT*_x_* NW, the investigations of the high‐resolution transmission electron microscope (HRTEM) and the selected area diffraction (SAED) pattern were performed. A MXene NW of a 3 µm length with high transparency indicates an ultrathin thickness of the E‐etched MXene as presented in Figure 2a. Additionally, Figure 2b reveals a clear crossed fringe associated from the crumbled and folded edge, which illustrates a superior flexibility of the MXene. From the observation of the lattice fringes, the *d*‐spacing of 0.343 nm accords with the reported value of the Nb_2_CT*_x_* NS.^[^ ^22^ ^]^ The SEAD of the E‐etched MXene sample is illustrated in Figure 2c. The diffraction rings demonstrate (421), (100), (102), and (201) planes of the Nb_2_CT*_x_* crystal,^[^ ^22^, ^39^ ^]^ which are indicated by the calculated *d*‐spacing of 0.21, 0.35, 0.181, and 0.128 nm, respectively. These patterns are coherent with PDF card #15‐0127 for hexagonal phase Nb_2_C of a space group of P63/mmc. The interlayer of 0.343 nm is comparable to the calculated value from a peak of 24.5° from the XRD pattern and corresponds to an interlayer of ≈0.35 nm. The atomic arrangement with a distinguished hexagonal array is shown by fast Fourier transform filtered HRTEM image (Figure 2d), evidencing the selective removal of the Al layer from its intrinsic MAX phase material and holding consistence with the SEAD image.

The SEM measurements show the cross‐sectional images of the Nb_2_CT*_x_* NS that restacked together due to the capillary effect (**Figure** **3**a). On the other hand, the NW holds an aggregation‐resistive ability which maintains a fluffy structure upon fast heating (Figure 3b). The Nb_2_CT*_x_* NW provided a large surface area and enhanced the electrocatalytic active site, which had an average length and width of ≈450 and 98 nm (Figure S2e,f, Supporting Information), respectively. Nonetheless, similar to the delaminated F‐functionalized MXenes, the HF‐free Nb_2_CT*_x_* is possibly sluggish from the inadequate ion accessibility to the surface/near‐surface of the MXene, lowering the double layer capacitance storage and ion intakes and thus decreasing the electrocatalytic ability. To alleviate from the restacking problem, a 3D backbone CFC was introduced to the Nb_2_CT*_x_* NS/NW, resulting in a 1D@3D networked hybrid structure (Figure 3c,d). The 3D‐Nb_2_CT*_x_* NS/NW structures were fabricated by dropcasting the MXene colloids (0.5 mg mL^−1^) on the surface of the CFCs. Upon the inward capillary force, the MXene dispersed in ethanol was rapidly evaporated with gentle heating (50 °C). With a stark contrast to the pristine CFC, the 1D/3D CFC owns a rougher surface as shown in the SEM images (Figure S3a,b, Supporting Information).

**Figure 3:**
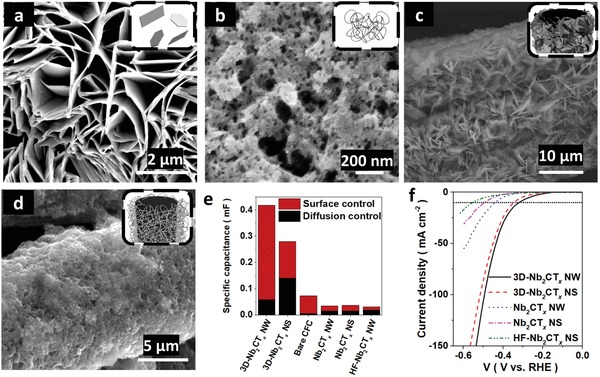
The structural, morphological, electrochemical kinetic electrocatalytic analysis and measurement for 1D/2D Nb_2_CT*_x_* MXene on 3D CFC backbones. a) SEM image for Nb_2_CT*_x_* NS and b) Nb_2_CT*_x_* NW c) SEM image for 3D‐Nb_2_CT*_x_* NS and d) 3D‐Nb_2_CT*_x_* NW. e) The contribution of the capacitive and diffusion‐controlled capacitance. f) The LSVs for Nb_2_CT*_x_* catalysts.

The boosted conductivity of the system was studied by the electrochemical impedance spectroscopy (EIS) measurement, as shown in Figure S3c, Supporting Information. A depressed semi‐circle indicates an enhanced surface area, and the high‐frequency tail with a higher slope depicts a lower Warburg resistance between the solid/liquid interface. The curves illustrate a strong aptitude of the efficient catalyst for fast ion‐transfer and migration of the electrolyte ions due to its porous surface. To quantify the enhancement effect of the surface area between Nb_2_CT*_x_* NW and NS, the MXenes were tested using N_2_ adsorption analyzer by BET method. The N_2_ absorption‐desorption isotherm and the pore size distribution showed an enhanced surface area with a mesoporous nature of Nb_2_CT*_x_* NW (Figure S3d, Supporting Information).^[^ ^4^ ^]^ The BET specific surface area of 75.6 m^2^ g^−1^ with a mesopore size of 3.29 nm was resulted from the aggregation of Nb_2_CT*_x_* NW, whereas the Nb_2_CT*_x_* NS suffered from a lower BET specific surface area of the 15 m^2^ g^−1^.

After the introduction of the 3D CFC backbones, the contact resistance is considerably reduced from 30 to 5 Ω, implying that the CFC not only acts as a backbone but also as an efficient current collector. According to the Tafel slope of Nb_2_CT*_x_* NW/NS (Figure S3e, Supporting Information), the HER activity occurred on the Volmer–Heyrovsky reaction rather than Tafel reaction. Under this electrochemical reaction, the activation energy of HER is sensitive to H_3_O^+^/e^−^ concentration and H coverage on the double layer of the catalysts,^[^ ^40^ ^]^ correlated to the surface‐ and diffusion‐controlled capacitance, respectively. It is worth noting that the electrochemically active surface area (ECSA, correlated to surface‐controlled capacitance) of the 3D‐Nb_2_CT*_x_* NW increased by about 14‐fold to the Nb_2_CT*_x_* NW and increased by fourfold to the summation of capacitance of bare CFC and Nb_2_CT*_x_* NW, where the increase in ECSA facilitates for HER activity. The enhancement in ECSA demonstrates a synergic effect between the 3D CFC backbones and MXene, generating more catalytic active sites for hydrogen production (Figure 3e).

Benefiting from the fast kinetics 1D structure, low internal resistance, and enlarged ECSA, the Nb_2_CT*_x_* NW and 3D‐Nb_2_CT*_x_* NW achieved a current density of 10 mA cm^−2^ on 440 and 322 mV, respectively (Figure 3f). HF‐etched Nb_2_CT*_x_* NW was fabricated to compare with the HF‐free MXene. Figure S3f and Table S2, Supporting Information, show HF‐etched Nb_2_CT*_x_* NW suffered from slow kinetics and high overpotential of 553 mV at a specific current density of 10 mA cm^−2^ in alkaline electrolyte. On the other hand, a large enhancement was observed in 0.5 m H_2_SO_4_ of reducing ≈150 mV and resulted in a lower overpotential of 396 mV at a specific current density of 10 mA cm^−2^. In the difference of overpotential and Tafel slope, HF‐free MXene manifests a relatively stable and superior performance in both acidic and alkaline electrolyte to HF‐etched MXene.

It is noted that this is the first to report for Nb_2_CT*_x_*, which is close to the theoretical overpotential of 0.4 V.^[^ ^32^ ^]^ The theoretical overpotential was deduced from the calculated Gibbs free energy for hydrogen adsorption (Δ*G*_H_) on Nb_2_CO_2_, a descriptor of intrinsic HER activity.^[^ ^41^ ^]^ The Tafel plot (Figure S3e, Supporting Information) holds a good agreement with the kinetic study, where the lowered slope is characteristic to the fast kinetics attributed to the high ion accessibility‐favored morphology and low contact resistance. To quantify the effect of cycling on the 3D‐Nb_2_CT*_x_* electrode, the electrocatalytic activity for HER and the change in the morphology and surface terminations were studied. On the one hand, the rigidly connected 3D foam‐like structure featured high robustness on the 3D‐Nb_2_CT*_x_* NW catalyst and kept a low overpotential over a 24 h period without apparent detachment as depicted in Figures S4 and S5, Supporting Information. After cycling, the surface of the electrode was attached to the K ions and the oxygen content was decreased. The increase of overpotential was probably due to an increase in the K terminations. On the other hand, 3D‐Nb_2_CT*_x_* NS and 3D‐HF‐etched Nb_2_CT*_x_* NW showed a relatively high and less stable overpotential over 24 h with some detachments in the SEM image.

With the high robustness and superior ion adsorption affinity toward metal ions,^[^ ^42^ ^]^ the 3D‐Nb_2_CT*_x_* NW allows a widespread application as hybrid systems. The atomically deposited metal ion rendered an enhanced HER/OER activity to the 3D‐Nb_2_CT*_x_* NW. The reduction of O—H binding strength and Δ*G*_H_ was attenuated by the introduction of transition metal (TM) ion.^[^ ^33^ ^]^ The TM‐ions grafted MXene electrodes were characterized by elemental mapping and XRD (Figures S6 and S7a, Supporting Information). The curve describes the TM ion being uniformly reacted with the surface —O terminations of the MXene as TM oxide at atomic scale. The enhancement on the electrocatalyst depends on the pristine MXene and the synergistic effect to the TM promotors. The connection is demonstrated in the LSVs of the TM@Nb_2_CT*_x_* NW (Figure S7b,c, Supporting Information). The benchmark (**Figure** **4**a) of the catalysts was followed by the order as regarded to the HER activity (Co^3+^ > Fe^3+^ > Ni^2+^) for both 1D and 1D@3D MXene‐CFC electrode system,^[^ ^14^ ^]^ whereas the TM@3D‐Nb_2_CT*_x_* NW catalysts exhibited overpotentials of 236, 302, and 365 mV at a specific current density of 10 mA cm^−2^ for the TM promotor of Co^3+^, Fe^3+^, and Ni^2+^, respectively. Correspondingly, a smaller Tafel slope is observed on the Co^3+^@3D‐Nb_2_CT*_x_* NW at higher current density region when compared to pure Pt plate (Figure 4b), demonstrating a fast kinetics process of the TM ion dispersed MXene catalysts. The superior electrochemical kinetics are comparable to the Pt/C.^[^ ^4^ ^]^ The large difference results from the kinetics barrier attributed to extra water dissociation process for Pt in alkaline electrolyte (Figure S3f, Supporting Information),^[^ ^43^ ^]^ while there is an absence of kinetics barrier to the TM metal species.^[^ ^44^ ^]^ Galvanostatic measurement on the Co^3+^@3D‐Nb_2_CT*_x_* NW exhibits a stable operation bias over 24 h, manifesting a good stability and durability for HER activity (Figure S7d, Supporting Information). The OER active Co^3+^ species rendered a good OER activity to the MXene which is comparable to the commercialized IrO_2_ and other catalysts (Table S2, Supporting Information),^[^ ^45^ ^]^ as shown in Figure 4c. Similar to the HER performance, the performance of the catalysts exhibited differently in the various architectures, suggesting the best comprehensive performance among the 3D catalysts. The good dispersity of Co^3+^ ions offered a significant enhancement to the pseudo‐capacitance of the Co^3+^@3D‐Nb_2_CT*_x_* NW catalyst and lowered the contact resistance of the system with a less resistive feature toward the high‐frequency tail region (Figure 4d). Such results open up a fascinating opportunity of constructing a hierarchical hybrid system by the conjugation of nanoparticles on the 1D MXene with 3D networks, and render innovative applications on sewage purification, and boost the performance on energy storage by the versatile method.^[^ ^14^, ^46^ ^]^

**Figure 4:**
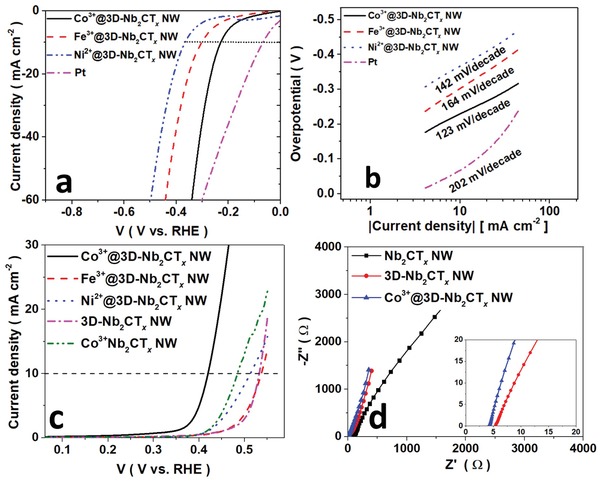
Electrochemical characteristics of Nb_2_CT*_x_* NW catalysts. a) Electrocatalytic performance on HER and its associated b) Tafel slope plot. c) The OER performance of the catalysis d) EIS of the various catalysts.

Proposed cathode reactions:

(2) $${\text{Nb}}_{2}{\text{CT}}_{x}+{\text{yZn}}^{2+}2{\text{ye}}^{-}⇌{\text{Nb}}_{2}{\text{Zn}}_{y}{\text{CT}}_{x}$$

A prototype of the home‐built flexible ZIB using 3D‐Nb_2_CT*_x_* NW as anode with an OCP of ≈0.6 V is presented in **Figure** **5**. Considering the advantages from the 1D NW at 3D backbones composite structure, the 3D‐Nb_2_CT*_x_* NW retains a high toughness during the ion intercalation process. Thus, the 3D‐Nb_2_CT*_x_* NW endows a long cycle life of 150 cycles and is distinct from the 2D MXene that the capacity drops to about half of their initial capacity upon the fast ion intercalation within 100 cycles (Table S3, Supporting Information).^[^ ^18^ ^]^ Figure 5b shows the capacity of the MXene battery increasing from 50 to 100 mAh g^−1^ at a current density of 300 mA g^−1^, demonstrating an activation effect of the MXene electrode. The flexible battery exhibited an energy density and power density of 140 Wh kg^−1^ and 420 W kg^−1^, together with high coulombic efficiency of 100%. The high retention and power delivery are comparable to organic‐electrolyte‐based MXene metal ion battery (Table S3). The activation effect of the 3D‐Nb_2_CT*_x_* NW based ZIB is also demonstrated in the CV curves as shown in Figure S8a, Supporting Information. Upon cycling, the peak current density of the MXene electrode is increased by twofold in both anodic and cathodic regions, while the 3D‐Nb_2_CT*_x_* NS cathode depicted a slight enhancement effect in the CV curve (Figure S8b, Supporting Information). The enhancement differences are interpreted by the decrease of cathode material (MXene) size and the fast kinetics of the 1D NW on the 3D backbone. Moreover, the difference in the zinc contents for 3D‐Nb_2_CT*_x_* NS and NW demonstrated the size effect on the zinc ion intakes (Figure S8c,d, Supporting Information). In summary, the architecture offers more active sites for the electrochemical reaction, facilitating Zn diffusion between surfaces, which is consistent with the previous discussion.

**Figure 5:**
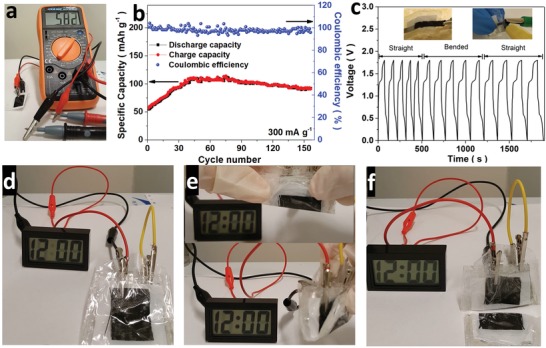
The performance of the flexible ZIB and deformation test for the battery. a) The open circuit potential (OCP) of the ZIB. b) The cycling capacities and coulombic efficiency of the Nb_2_CT*_x_* NW electrode over 200 cycles at a current density of 300 mAg^−1^. c–f) The bending and cutting test on a pair of flexible MXene‐based ZIB, testing on a charge/discharging current density of 2 A g^−1^. The liquid crystal display (LCD) clock can be lighted up by a bias of 1.1 V.

To investigate the enhancement in the Zn^2+^ ion intakes, the ex situ XRD was investigated as shown in Figure S9a, Supporting Information. Prior to the cycling test, the MXene cathode shows a fine interspacing peak with a comparable ratio to the original MAX phase peak. When the battery was charged/discharged after 100 cycles, the formation of the upshifted peak appears at 8.11° and a small peak at 7.05° representing the intercalation of Zn^2+^ ions. When the battery was discharged to 0.3 V, the peak further upshifts from 7.05° to 6.80° when the battery was charged and discharged at 1.5 and 0.3 V, respectively. The 2D XRD also manifests an intense spectrum line in the low angle region, indicating a high crystallinity of the MXene electrode of the charged state (Figure S9b, Supporting Information). In agreement to the XRD peak upshift from 11.6° to 8.11°, it is proposed that the large activation effect was resulted from the enlargement of the interlayer spacing from 1.53 to 2.18 nm, and the shortened diffusion length between cathode materials.^[^ ^3^ ^]^ The galvanic charge/discharge (GCD) curves show a plateau at 0.7–0.8 V with a good agreement to the Zn/Zn^2+^ reversible potential of 0.76 V (Figure S10a,b, Supporting Information). The pseudocapacitive contribution at different scan rates is calculated by the convolution from the CV curves and the equations in Supporting Information. Figure S10c, Supporting Information, depicts the calculated ratio of the capacitance contribution for the diffusion control and surface (capacitive) control component. A clear trend of increasing surface control contribution is shown by the gradual increase from 14% to 59% as the scan rates decrease, indicating a merit of shorten diffusion length in the MXene NW.^[^ ^47^ ^]^ Taking advantage of high surface‐controlled capacitance, the 3D‐Nb_2_CT*_x_* NW demonstrated a feasible rate capacity for different current densities (Figure S10d, Supporting Information). To demonstrate the stability of the flexible battery under deformation situation, GCD curves were measured during the folding condition (Figure 5c). The flexible battery exhibited superior stability during the folded and unfolded process with a constant operation potential, similar to the other carbon‐based flexible electrode.^[^ ^48^ ^]^ Furthermore, the battery exhibited desirable stability during the test without any significant change in the potential and continuously powered up a liquid crystal display (LCD) clock during the deformation condition as depicted in Figure 5d–f. Under an extreme condition, the flexible battery was cut into half with non‐toxic/flammable electrolyte leakage and the battery was still able to turn on the LCD clock. These tests show the emerging application and possibility of the 3D‐MXene electrode for flexible ZIB in a vast wearable device.

In conclusion, we report a HF‐free method capable of synthesizing 1D Nb_2_CT*_x_* in 4 h with dilute HCl electrolyte for the first time. A 1D interconnected 3D network on a CFC flexible substrate is successfully developed, in which the introduction of the CFC backbone not only acts as a framework for the NW but also enhances the ion accessibility hence boosting the electrochemical performance of MXene related compounds. By the optimization of coupling with the TM metal ions, the TM/3D‐Nb_2_CT*_x_* NWs demonstrate a surpassing electrocatalytic performance with an extremely low overpotential comparable to the best existing metal‐based electrocatalyst. Furthermore, the flexible MXene compound based cathode is capable of delivering a high‐power density of 420 W kg^−1^. The simple and toxic‐free synthesis method in this work provides an emerging route toward fabricating different nanoscale forms of MXene composition. Our work demonstrates a rational designed architecture of MXene composition based NW on 3D CFCs and reveals the prominent energy conversion and storage performance of HF‐free MXene. It is envisioning that the strategy proposed in this work can be expanded to the other application fields using 1D nanowires and 1D/3D structures.

## Experimental Section

##### Materials and Reagents

Niobium aluminum carbide (Nb_2_AlC, 200 mesh, 99% purity) was purchased from Laizhou Kai Ceramic material Co., Ltd. Hydrochloride, potassium hydroxide, lithium fluoride, and sodium hydroxide were purchased from Sigma. Cobalt nitrate hexahydrate, iron (III) nitrate nonahydrate, and nickel nitrate hexahydrate were purchased from Macklin Biochemical Co., Ltd. Polyvinyl alcohol (88%) was purchased from Aladdin‐reagent Co., Ltd. All materials were used as received without further purification.

##### Purification of Carbon Fiber Cloths

The CFCs were purified as reported previously.^[^ ^14^, ^31^ ^]^ The CFCs were first cleaned by ethanol and acetone and immersed into the HNO_3_ solution for 6 h under reflux. The CFCs were neutralized by the NaOH solution, followed by water wash for several times and dried in an oven at 60 °C.

##### Synthesis and Collection of the 1D/2D MXene

The hydrolyzed Nb_2_AlC was first fabricated as a precursor for the MXene NW. In the typical route, 1 mL of 6 m KOH solution was added to 100 mg Nb_2_AlC followed by intense stirring under heating at 50 °C for 4 h. MXenes were synthesized by the HF‐free route^[^ ^14^ ^]^ via 3D electrode preparation. The as‐diminished MAX phase with the carbon black mixture in a ratio of 95:5 was directly dispersed in 1% PVA and uniformly dropcasted onto the CFC substrate. By the anodization to the Al layer under 1 V for 4 h at 0.5 m HCl electrolyte, the Al layer was selectively removed. For the 2D MXene, the procedure was similar but no hydrolyzation step was required for the precursor. The as‐synthesized MXenes were placed in a test tube for 4 h. 80% of the supernatant was subsequently collected by pipette, and the residues were discarded. The 1D MXene was purified by collecting the supernatant which was obtained from centrifugation at 4000 rpm. for 5 min. To remove the presence of potential carbide‐derived carbon, the supernatant was centrifuged at 9000 rpm and washed by D.I. water for several times.

##### Preparation of HF‐Etched Nb*_2_*CT*_x_* NW

For the fabrication of HF‐etched Nb_2_CT*_x_* NW, Nb_2_AlC powder (100 mg) was immersed in a mixture of LiF and HCl (1:10 w*/*w%) at 55 °C for 50 h under stirring, as previously described.^[^ ^13^ ^]^ Afterward, the MXene suspension was repeatedly washed by D.I. water and centrifuged at 9000 rpm until the pH value achieved to ≈7. After the purification process of the MXene, the NW was separated by a similar process to the HF‐free MXene NW.

##### Preparation of Co^3+^/Fe^3+^/Ni^2+^@Nb_2_CT*_x_* NW

All catalysts were fabricated by a similar approach, as reported previously. MXenes (1 mg) were well dispersed in 2 mL of NaOH solution (1 m) and intensely stirred by magnetic bars for 1 h. The TM metal seed was mixed with the NaOH‐MXene followed by several times of wash by the D.I. water.

##### Dropcasting of Nb_2_CT*_x_* NW/TM Metal Dispersed Nb_2_CT*_x_* NW/NS on 3D CFC Backbone

The as‐synthesized MXene colloids were dispersed in 0.5 mL mixture of water and alcohol (in a ratio of 1:1). The CFCs were heated at 50 °C in air. Afterward, 0.2 mL of MXene colloid was transferred by pipette and then uniformly dropcasted on the porous CFC. Upon gentle heating, the nanowire networks were formed gradually layer by layer by repeating the dropcasting procedure several times.

## Conflict of Interest

The authors declare no conflict of interest.

## Supporting information

Supporting Information
