# Supplementary material for: Efficient Energy Conversion and Storage Based on Robust Fluoride‐Free Self‐Assembled 1D Niobium Carbide in 3D Nanowire Network
Source: Adv Sci (Weinh). 2020 Apr 6;7(10):1903680. doi: 10.1002/advs.201903680 (PMC7237850; doi:10.1002/advs.201903680)
Supplement: Supplementary file 1 — Supporting Information [file ADVS-7-1903680-s001.pdf]

Copyright WILEY-VCH Verlag GmbH & Co. KGaA, 69469 Weinheim, Germany, 2020.

ADVANCED SCIENCE
Open Access

Supporting Information

for *Adv. Sci.,* DOI: 10.1002/advs.201903680

Efficient Energy Conversion and Storage Based on Robust Fluoride-Free Self-Assembled 1D Niobium Carbide in 3D Nanowire Network

*Sin-Yi Pang, Weng-Fu Io, Lok-Wing Wong, Jiong Zhao, and Jianhua Hao**

Supporting Information

Efficient energy conversion and storage based on robust fluoride-free self-assembled 1D niobium carbide in 3D nanowire-network

Sin-Yi Pang[1], Weng-Fu Io[1], Lok-Wing Wong[1], Jiong Zhao[1] and Jianhua Hao[1,*]

[1]Department of Applied Physics, The Hong Kong Polytechnic University, Hong Kong, P. R. China

*Corresponding author. Email: jh.hao@polyu.edu.hk

Material characterization

X-ray photoelectron spectroscopy (XPS) was carried out by Thermo Scientific™ Nexsa™ X-Ray photoelectron spectrometer system, coupled to monochromatic and microfocus Al K_α X-ray source (1486.2 eV). High-resolution XPS spectra were collected by double-focusing hemispherical analyzer with 180° angle. Powder X-ray diffraction (XRD) patterns of MXene were measured using a Rigaku smart lab 9 kW (Rigaku, Japan) equipped a 2D detector with Cu K_α radiation (λ = 0.154 nm). Morphology of the Nb_2CT_x NW sample was taken using TEM (JEOL 2100F Transmission Electron Microscope, Japan) with 200 kV accelerating voltage, while the exposure time is 0.5 s for requesting the HRTEM images. Elemental characterizations and the morphologies of the catalysts were investigated by scanning electron microscope (SEM, JEOL Model JSM-6490) that equipped with an energy-dispersive X-ray (EDX) spectrometry system. Raman spectra of the samples were obtained from a Witec Confocal Raman system, paired with an excitation source of continuous-wave (C.W.) 532 nm diode laser of 1 mW laser power. The specific surface areas of the catalysts were acquired using surface area and porosity analyzer (ASAP 2020, micromeritics, USA), while a determination method of N_2 adsorption on catalysts by Brunauer–Emmett–Teller (BET) method was used at an analyzing temperature of -195.8°C (~77 K). To avoid the signal error arising from adsorbed water and organic compounds, pretreatment was conducted by heating the catalysts in vacuum at 200 °C (2h).

HER and OER measurement

The electrochemical characterizations were evaluated by using Solartron Electrochemical workstation with standard three electrode system in 1 M KOH (aq). A polished glassy carbon (GC) electrode was served as working electrode while a carbon rod and a standard calmer electrode filled with saturated KCl solution were used as counter and reference electrode, respectively. The MXene colloid was dispersed in 0.5 mL D.I. water and drop-casted onto GC electrode without adding extra binder and conductive polymer. The mass loading on the working electrode was 0.1 mg cm^{-2} for all catalysts. The LSVs for HER and OER were recorded at a scan rate of 5 mV s^{-1} without external gases purging.

Cell fabrication and test

The electrochemical test of the MXene zinc ion battery was carried out by a home-built flexible battery. The cathodes were prepared by drop-casting method using the certain amount of MXene colloid (0.5-2mg/mL) onto a carbon fiber clothes without any additional additives were added. The CFC electrode was dried at 50°C for 3 h in an ambient air. High purity zinc foil (>99.9%) as cut into comparable size to the CFC electrode. PVA films were utilized as the protective battery shell and the flexible backbones. Microporous membrane (Celgard 2325) with a pore size of 3 nm and 25 µm was used as the separator and 2 M $ZnSO_4$ (aq) was filled in the PVA sealed flexible battery. Electrochemical measurement of cycling voltammetry, galvanic charge/discharge and electrochemical impedance spectroscopy experiments were recorded at Solartron Electrochemical workstation. A potential window of 1.45 V ranging from 0.2 V to 1.73 V for CV and GCD measurement, while the EIS curve was swept from 1 MHz to 10 mHz with a voltage amplitude of 4 mV vs. open circuit voltage (OC).

Supporting tables

Table S1. Quantitative EDX results with different etching voltage.

**Material**	**Etching Voltage (V *vs*. RHE)**	**Nb At%**	**Al At%**	**O At%**	**C At%**	**Nb/Al**
Nb_2CT_x/Nb_2AlC	0.5	11.4	4.81	5.24	78.55	2.37
	1.0	11.99	3.85	5.2	78.96	2.72
	1.2	11.52	4.41	7.16	76.91	2.61

Table S2. A comparison of Nb_2CT_x electroanalytic with recently reported works in HER/OER performance.

Catalyst	Electrolyte	b@$_{J=10\ mA\ cm^{-2}}$ (mV dec^{-1})	η@$_{J=10\ mA\ cm^{-2}}$ (mV)	Reference
**HER**				
**Nb_2CT_x NS**	**1M KOH**	**131**	**507**	**This work**
**Nb_2CT_x NW**	**1M KOH**	**120**	**441**	**This work**
**Nb_2CT_x NW**	**0.5 M H_2SO_4**	**178**	**342**	**This work**
**3D-Nb_2CT_x NS**	**1M KOH**	**106**	**350**	**This work**
**3D-Nb_2CT_x NW**	**1M KOH**	**110**	**322**	**This work**
**Co^{3+}@ 3D-Nb_2CT_x NW**	**1M KOH**	**120**	**236**	**This work**
**HF-etched Nb_2CT_x NW**	1M KOH	226	553	
**HF-etched Nb_2CT_x NW**	0.5 M H_2SO_4	154	396	This work (as a reference)
**Pure Pt**	0.5 M H_2SO_4	50	77.3	
**Pure Pt**	1M KOH	79.3	142	
**Ti_2CT_x**	0.5 M H_2SO_4	127	540	*J. Am. Chem. Soc.* **2019**, 141, 9610-9616
**MXene nanofiber**	0.5 M H_2SO_4	97	169	*ACS Sustainable Chem. Eng.* **2018**, 6, 8976−8982
**Ti_2C**	0.5 M H_2SO_4	169	609	*ACS Energy Lett.* **2016**, 1, 589−594
**Mo_2C**	0.5 M H_2SO_4	74	305	
**OER**				
**Co^{3+}@ 3D-Nb_2CT_x NW**	1 M KOH	61.8	420	**This work**
**Co^{3+}-Ti_2CT_x**	1 M KOH	63.5	425	*J. Am. Chem. Soc.* **2019**, 141, 9610-9616
**Co/N-CNTs@$Ti_3C_2T_x$**	0.1 M KOH	79.1	411	*Adv. Mater. Interfaces* **2018**, 5, 1800392
**$Ti_3C_2T_x$-CoBDC**	0.1 M KOH	48.2	410	*ACS Nano* **2017**, 11, 5800-5807
**IrO_2**	0.1 M KOH	60.8	460	
**CoP@Ti_3C_2**	1 M KOH	51	290	*ACS Nano* **2018**, 12, 8017-8028
**CoP**	1 M KOH	81	303	

Where η represented overpotential and b corresponded to the Tafel slope.

Table S3. A comparison of Nb_2CT_x electroanalytic with recently reported works in metal ion battery.

Cathode material	Battery type	electrolyte	Current density	Capacity	Retention (cycle life)	Reference
**3D-Nb_2CT_x NW**	**Zinc ion battery**	**2 M $ZnSO_4$**	**300 mA g^{-1}**	**100 mAh g^{-1}**	**150 cycle, ~100%**	**This work**
Cr_2CT_x	Zinc ion battery	2 M $ZnSO_4$	50 mA g^{-1}	100 mAh g^{-1}	100 cycle, 98%	*J. Am. Chem. Soc.* **2019**, 141, 9610-9616
Nb_2CT_x	Lithium ion battery	1M LiPF6	10 C	110 mAh g^{-1}	150, ~83%	*J. Am. Chem. Soc.* **2013**, 135 15966-15969
V_2CT_x			10 C	125 mAh g^{-1}	150, ~67%	
V_2CT_x	Aluminum ion battery	$AlCl_3$/ [EMIm]Cl	300 mA g^{-1}	150 mAh g^{-1}	100 cycle, ~50%	*ACS Nano* **2017**, 11, 11135-11144

Supporting Figures

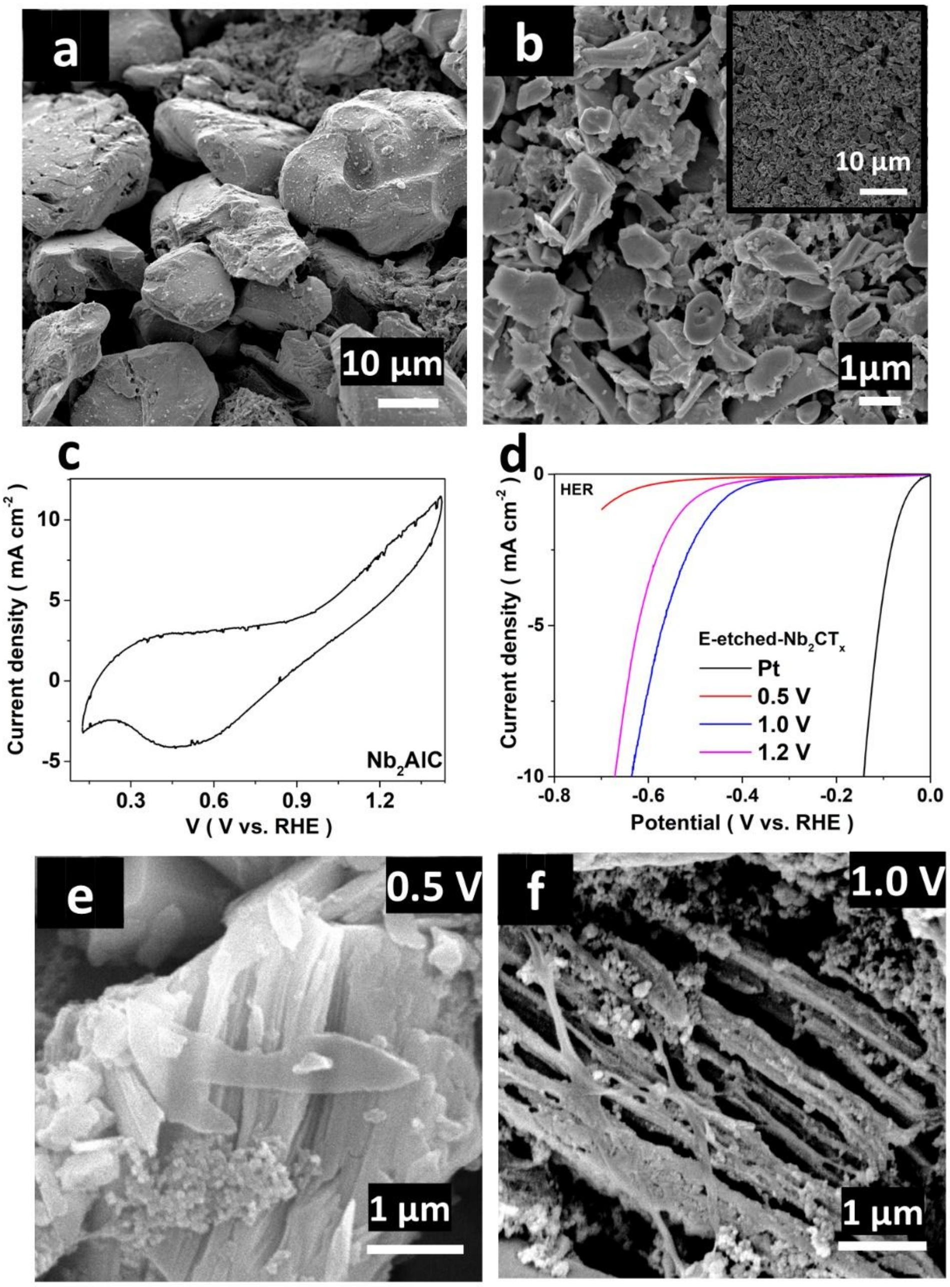

Figure S1. SEM images and electrochemical analysis of the precursors and MAX/MXene composites. MAX phase precursor for (a) Nb_2CT_x NS and (b) Nb_2CT_x NW. (c) Cyclic voltammograms on Nb_2AlC MAX phase material, indicating two-stage etching reaction. (d) Linear sweep voltammetry shows a reducing overpotential as the etching voltage

is near to the first stage reaction peak. SEM images of Nb_2CT_x produced from different E-etching voltage of (e) 0.5V and (b) 1.0 V.

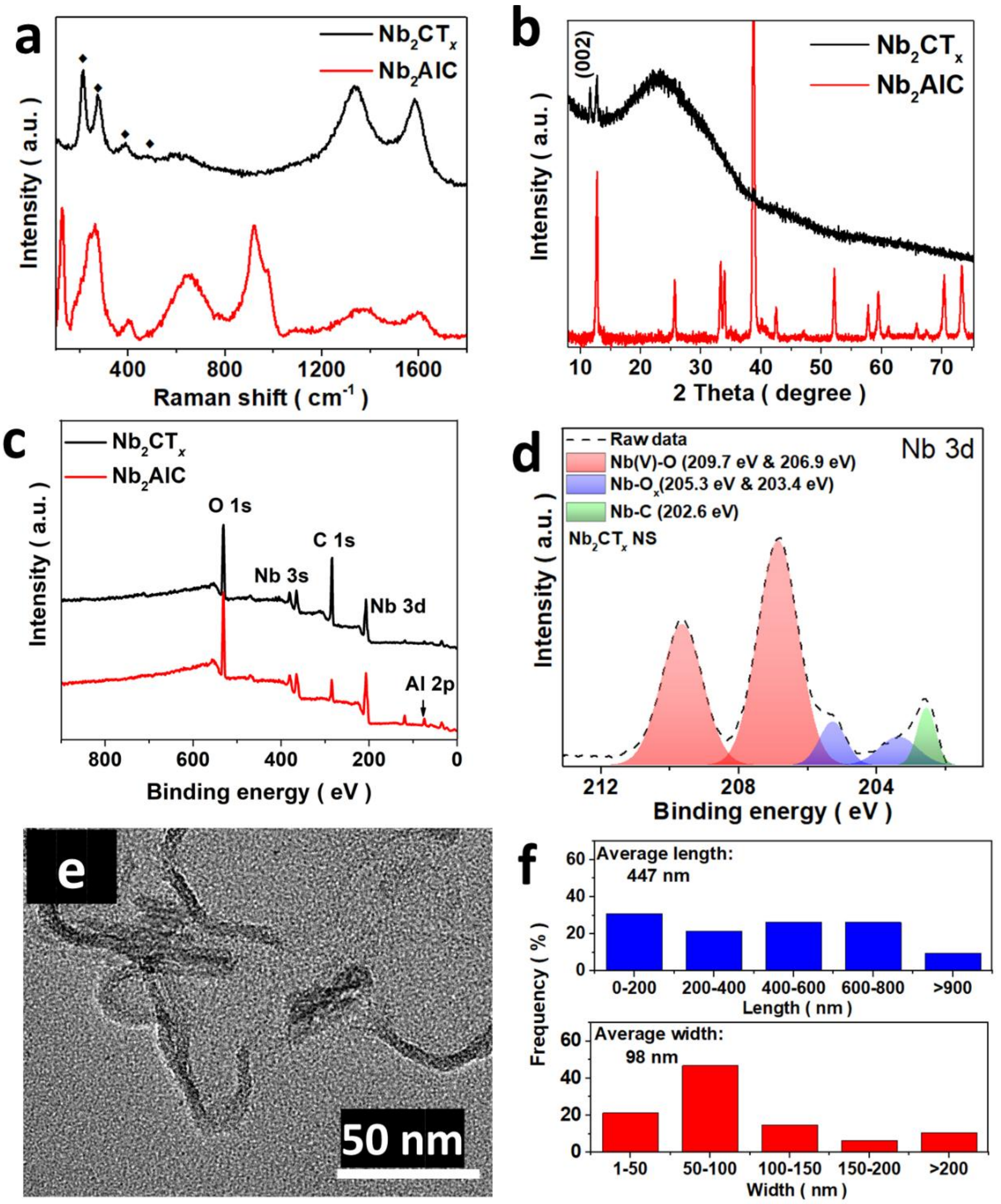

Figure S2. Structural characterization of Nb_2CT_x nanowires. (a) Raman spectroscopy and (b) X-ray diffraction pattern confirm the selective etching of the MXene from the MAX phase materials. (c) X-ray photoelectron spectroscopy (XPS) survey and (d) high-resolution deconvoluted spectrum of Nb_2CT_x on Nb 3d. (e) TEM image of Nb_2CT_x nanowires and (f) the statistical survey of nanowires from 50 samples.

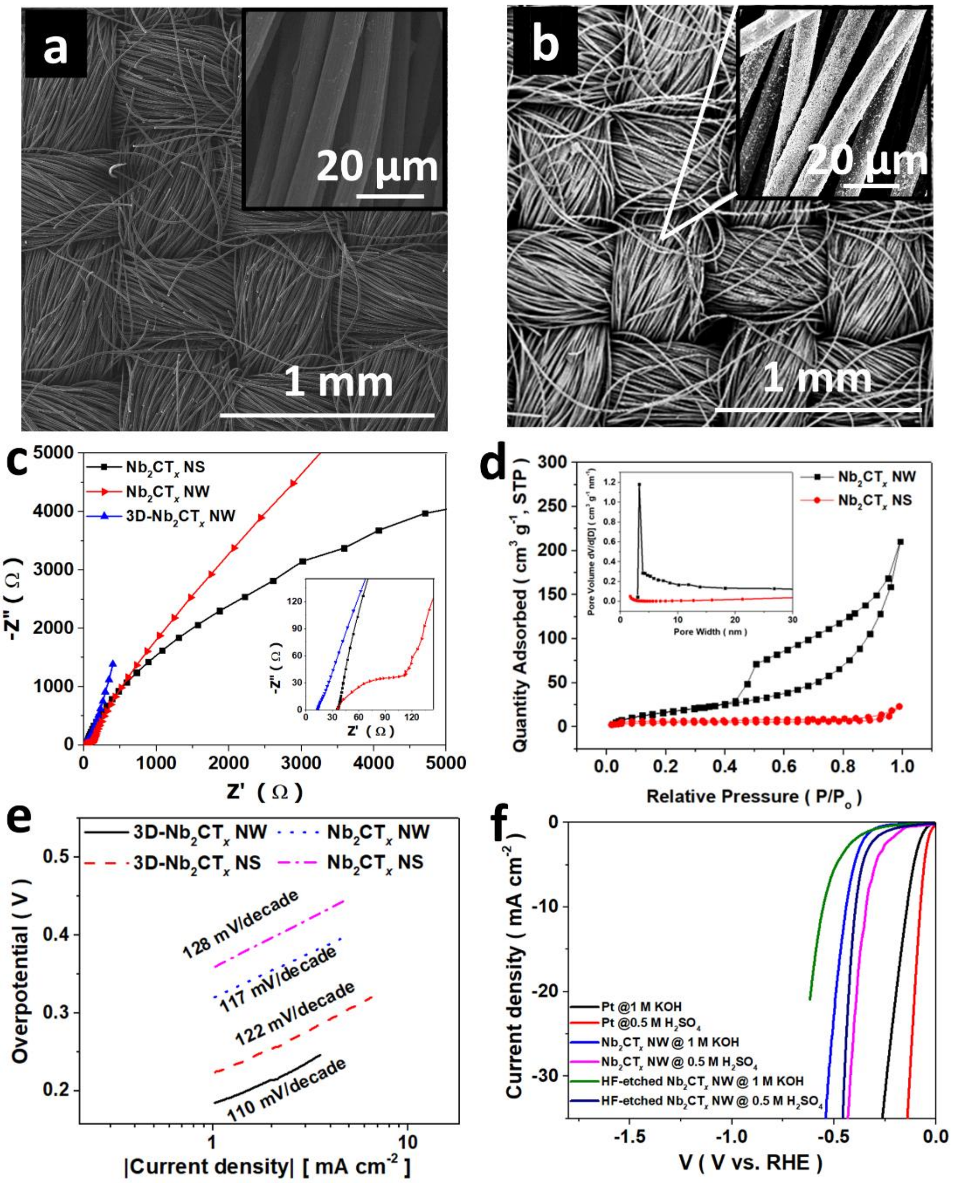

Figure S3. **The morphology, porosity and electrochemical study of the pristine CFC substrate and the 3D MXene catalysts**. The comparison of (a) bare CFC and (b) 3D-Nb_2CT_x NW@CFC. (c) The electrochemical impedance spectroscopy scanned from 10 MHz to 1 mHz and the Tafel slope plot for the catalysts. (d) N_2 adsorption-desorption isotherm and pore size distributions of Nb_2CT_x NW and Nb_2CT_x NS. (e) Tafel slope of Nb_2CT_x electrocatalysts. (f) LSV of Nb_2CT_x NW, HF-etched Nb_2CT_x NW and Pt plate in acidic and alkaline solution.

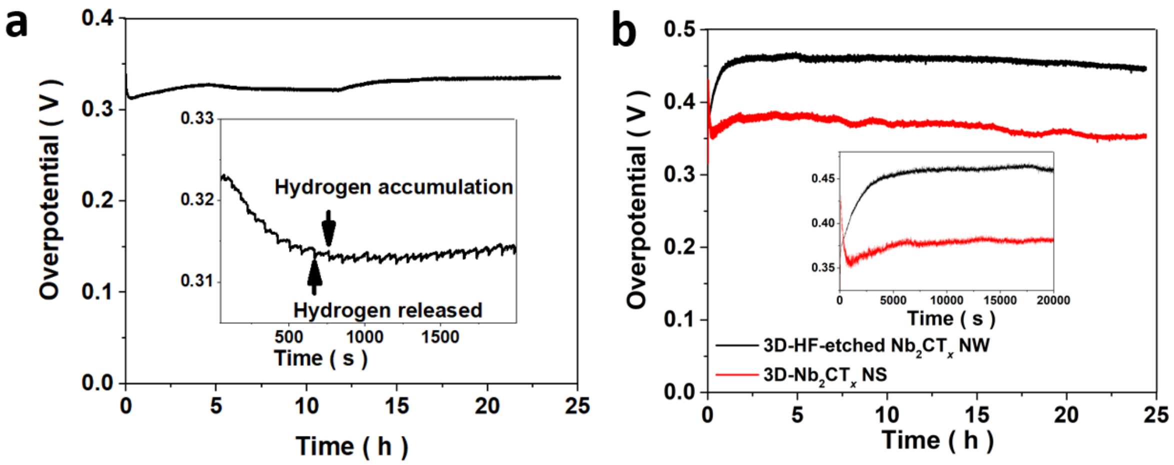

Figure S4. Stability test of the 3D-Nb_2CT_x NW/NS and 3D-HF-etched-Nb_2CT_x NW electrocatalysts. (a) The measurement of 3D-Nb_2CT_x NW performed in characterizing the chronopotential response for 24 h at a fixed current density of 10 mA cm^{-2}, demonstrating a robust layer to layer structure and high stability to the catalyst. (b) Stability test for 3D-Nb_2CT_x NW and 3D-HF-etched-Nb_2CT_x NW for 24 h at a fixed current density of 10 mA cm^{-2}.

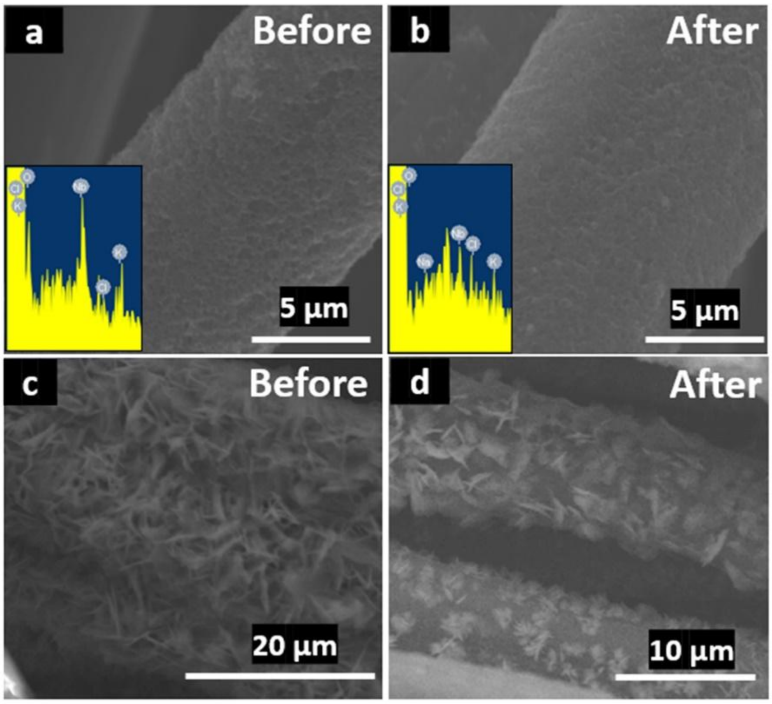

Figure S5. The morphology comparison and the elemental analysis of the 3D-Nb_2CT_x electrocatalysts after the stability test. The SEM shows that 3D-Nb_2CT_x NW electrode maintains (a-b) a stable structure, whereas (c-d) 3D-Nb_2CT_x NW electrode appears detachment after 24 h hydrogen release process.

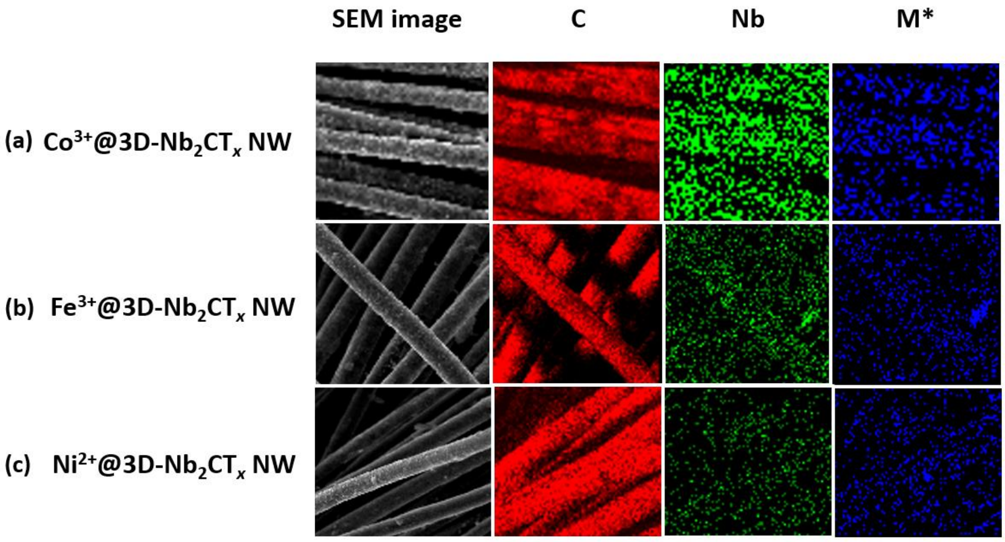

Figure S6. SEM and elemental mapping analysis for the various catalysts. (a) Co^{3+}@3D-Nb_2CT_x NW (b) Fe^{3+}@3D-Nb_2CT_x NW and (c) Ni^{2+}@3D-Nb_2CT_x NW with carbon, niobium and their corresponded TM metal (M*) elemental mapping.

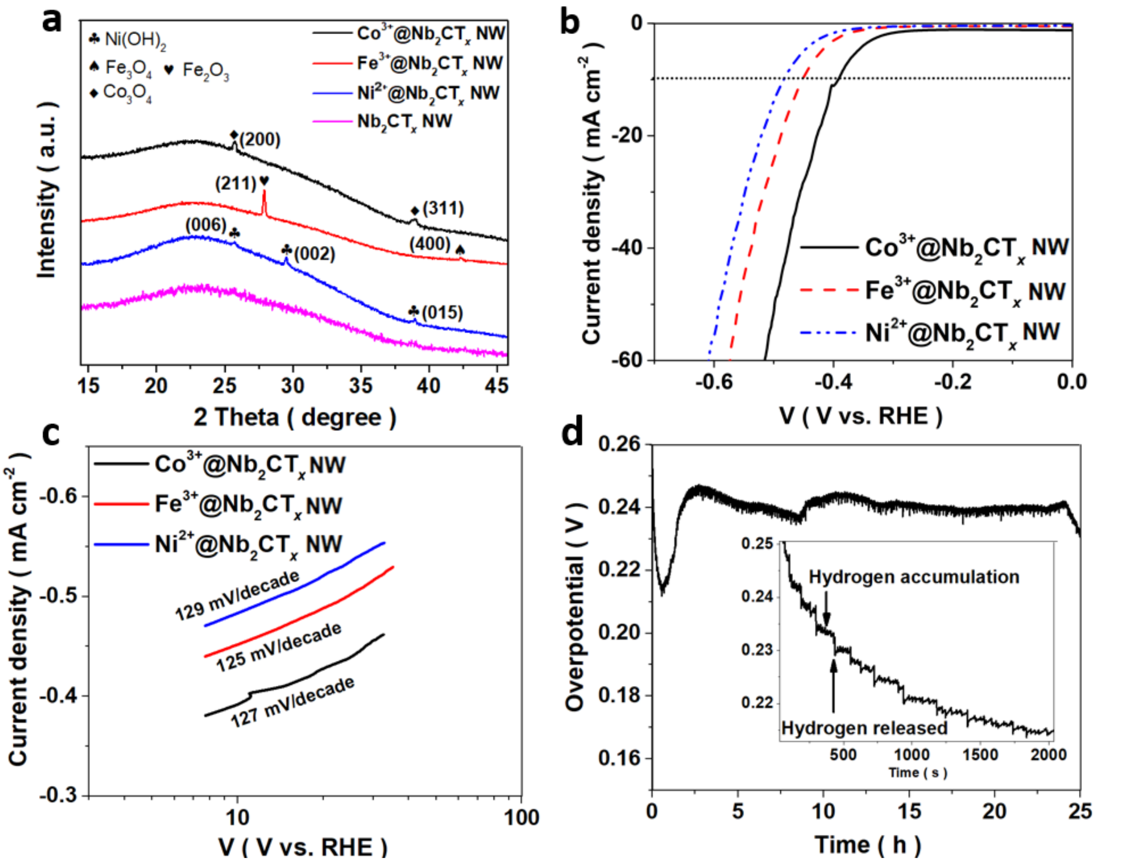

Figure S7. The structural and electrochemical characterizations of the TM metal doped on 3D-Nb_2CT_x NW electrocatalyst. (a) XRD spectrum for the various catalysts. (b) The LSV and the corresponded (c) Tafel slope for the electrocatalyst. (d) The stability test shows the solid electrocatalytic performance of the Co^{3+}@3D-Nb_2CT_x NW.

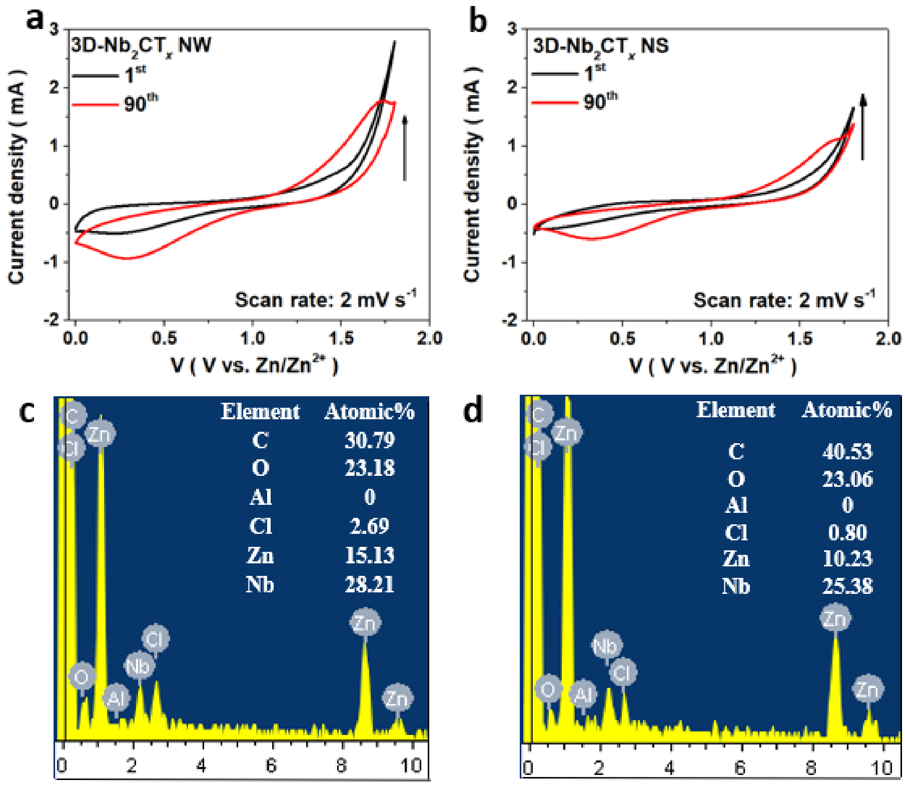

Figure S8. The capacity-related electrochemical characterization and elemental analysis. The CV curve shows the capacity was increased upon the activation process on (a) 3D-Nb_2CT_x NW and (b) 3D-Nb_2CT_x NS. The EDX results of (c) 3D-Nb_2CT_x NW and (d) 3D-Nb_2CT_x NS at discharge stage (0.3 V).

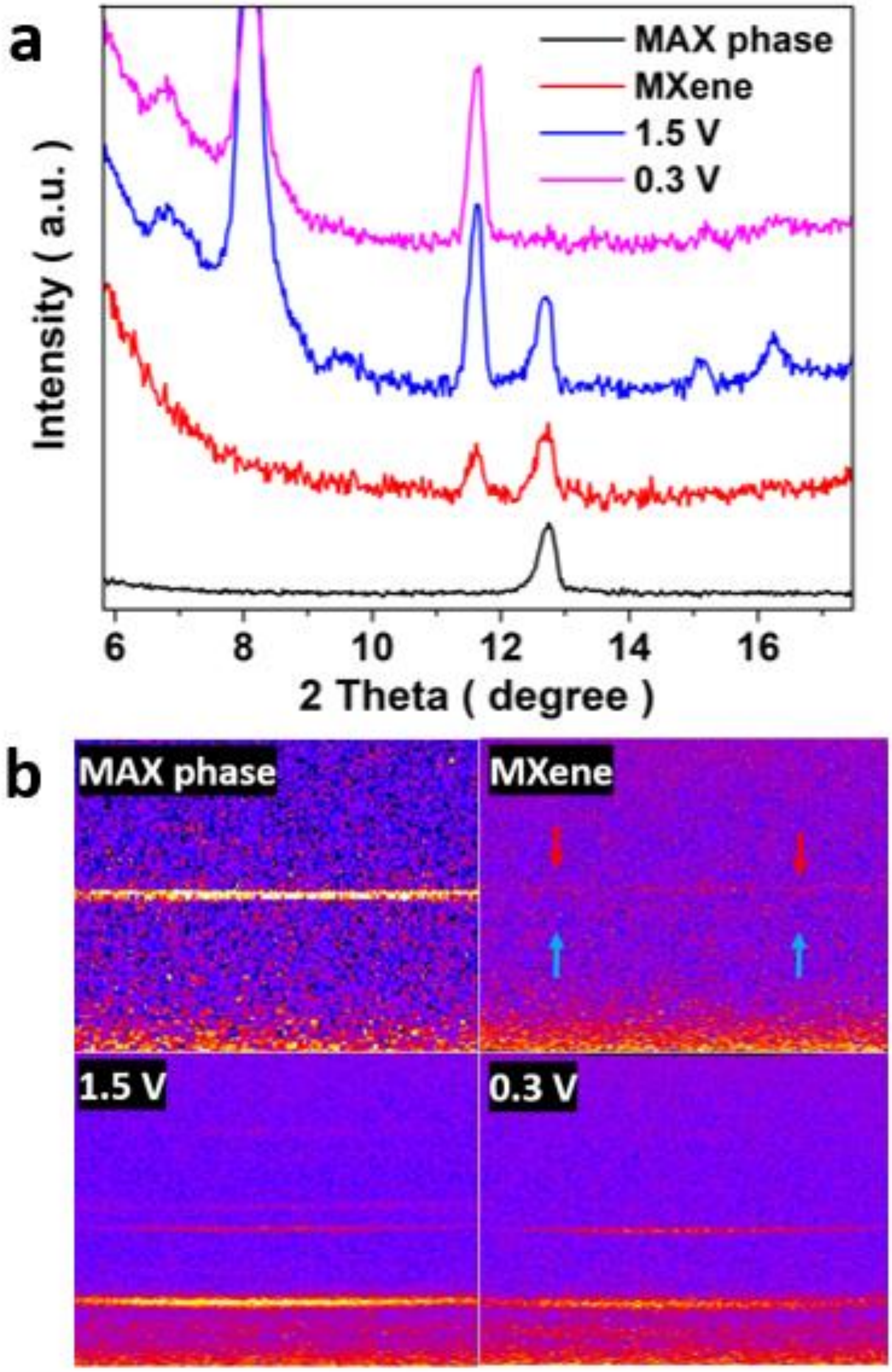

Figure S9. The structural characterization of the 3D-Nb2CTx NW in battery application. The XRD curve for the battery in difference charging/discharging state. The formation of the new peak on the low angle indicates the lubrication of the ion accessing channel and result in an increase capacity. (b) 2D XRD at different charging state. The intense spectrum line indicates high crystallinity on the corresponded peaks.

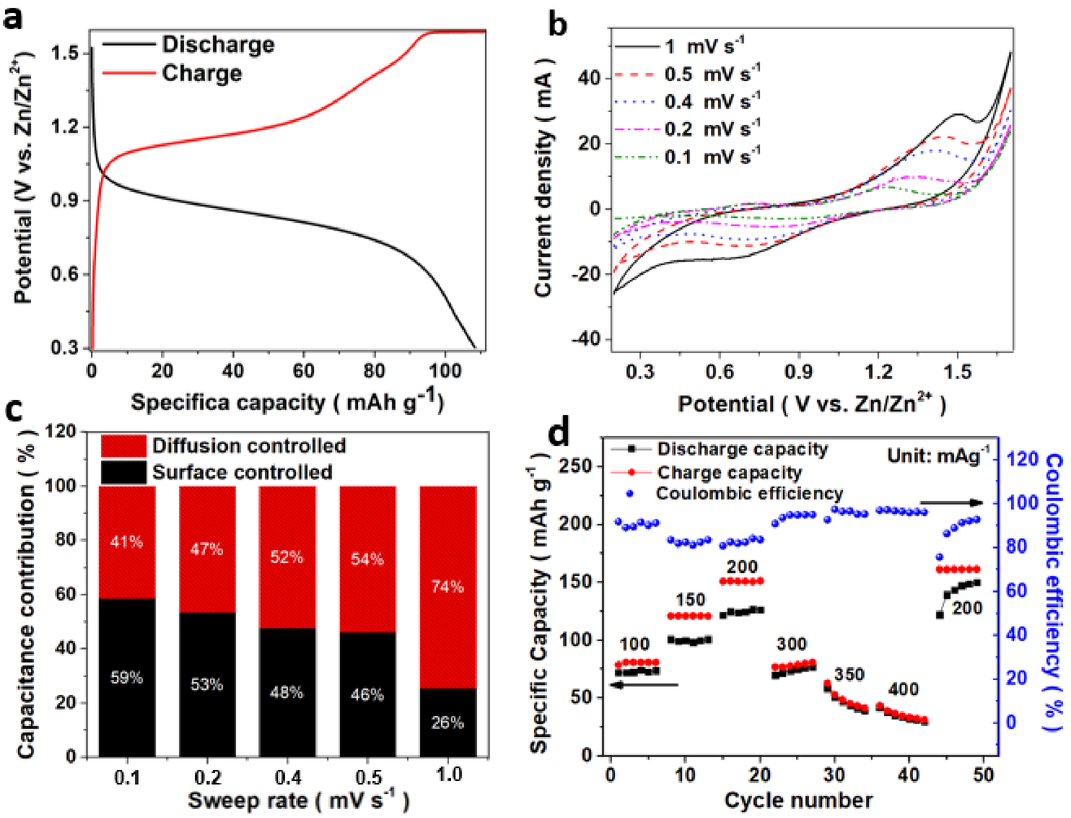

Figure S10. The electrochemical properties of the 3D-Nb_2CT_x NW. (a) Charge/discharge cycles for the 3D-Nb_2CT_x NW cathode at a current density of 150 mAg^{-1} (b) CV curve for 3D-Nb_2CT_x NW at different sweep rate ranged from 0.1 to 1 mV s^{-1}. (c) The electrochemical kinetics analysis for 3D-Nb_2CT_x NW in a battery system. (d) Rate capacity and retention performance for the flexible battery at different charge/discharge current densities.
